# Nuclear expression of lysyl oxidase enzyme is an independent prognostic factor in rectal cancer patients

**DOI:** 10.18632/oncotarget.9623

**Published:** 2016-05-26

**Authors:** Na Liu, Thomas R. Cox, Weiyingqi Cui, Gunnar Adell, Birgitta Holmlund, Jie Ping, Ingvar Jarlsfelt, Janine T. Erler, Xiao-Feng Sun

**Affiliations:** ^1^ Department of Oncology and Department of Clinical and Experimental Medicine, Linköping University, SE-58185, Linköping, Sweden; ^2^ State Key Laboratory of Cancer Biology and Xijing Hospital of Digestive Diseases, Xijing Hospital, Fourth Military Medical University, 710032, Xi'an, China; ^3^ Biotech Research and Innovation Centre (BRIC), University of Copenhagen, DK-2200, Copenhagen, Denmark; ^4^ Shanghai Center for Bioinformation Technology, 201203, Shanghai, China; ^5^ Department of Pathology, Ryhov Hospital, SE-55111, Jönköping, Sweden

**Keywords:** lysyl oxidase, nuclear localisation, prognosis, rectal cancer patient

## Abstract

Emerging evidence has implicated a pivotal role for lysyl oxidase (LOX) in cancer progression and metastasis. Whilst the majority of work has focused on the extracellular matrix cross-linking role of LOX, the exact function of intracellular LOX localisation remains unclear. In this study, we analysed the LOX expression patterns in the nuclei of rectal cancer patient samples and determined the clinical significance of this expression. Nuclear LOX expression was significantly increased in patient lymph node metastases compared to their primary tumours. High nuclear LOX expression in tumours was correlated with a high rate of distant metastasis and increased recurrence. Multivariable analysis showed that high nuclear LOX expression was also correlated with poor overall survival and disease free survival. Furthermore, we are the first to identify LOX enzyme isoforms (50 kDa and 32 kDa) within the nucleus of colon cancer cell lines by confocal microscopy and Western blot. Our results show a powerful link between nuclear LOX expression in tumours and patient survival, and offer a promising prognostic biomarker for rectal cancer patients.

## INTRODUCTION

Colorectal cancer (CRC) is the third most common cancer worldwide with approximately 694,000 estimated deaths. This makes it the fourth most common cause of cancer death [1]. Although the treatment of CRC has been improved significantly during the past two decades, the 5-year survival rate has improved only marginally [2]. Local and distant recurrence still occur in one third of patients with advanced stage (T3 or T4) resectable rectal cancer [3]. Therefore, novel molecular biomarkers for the identification of patients at high risk of regional relapse and distant metastasis are urgently required to further refine patient treatment regimens.

Lysyl oxidase (LOX) is a secreted copper-dependent amine oxidase, which catalyses the crosslinking of collagens and elastin in the extracellular matrix (ECM) and is essential for embryonic development and would healing. It is synthesized as a 50-kDa proenzyme, secreted, and processed into a 32-kDa mature, active enzyme and an 18-kDa propeptide (LOX-PP) [4]. In addition to an ECM remodeling role, LOX has also been shown to function intracellularly, regulating both cell signalling and gene expression [5, 6].

However, the precise role of LOX in cancer remains controversial. Initially it was identified as a tumour suppressor. Ectopic LOX expression was found to inhibit Ras-mediated transformation, and decreased LOX expression has been reported in several types of cancers [4, 7, 8]. In contrast, recent publications have demonstrated the overexpression of LOX in many types of solid cancer, including brain [9], head and neck [10], oral and oropharyngeal squamous cell [11], breast [12] and ovarian cancer [13]. LOX has been shown to promote cancer cell proliferation, metastasis and angiogenesis [14, 15], supporting a role for LOX as a tumour and metastasis promoter. One explanation for the seemingly paradoxical role of LOX in cancer is likely the existence of multiple forms and differential localisation of LOX. Increasing evidence indicates that the tumour suppressor activity of LOX lies in the cleaved LOX-PP (18-kDa) which can re-enter the nucleus following extracellular cleavage from the mature enzyme and repress oncogenes such as bcl-2 [4, 16–18]. On the contrary, the secreted LOX mature enzyme is typically reported to increase ECM stiffness, activate oncogenic signalling pathways and play a tumour promoting role [19, 20].

Given the paradoxical role of LOX in cancer and the potential link to specific localisation, more detailed studies are required to determine the relationship between its patterns of expression and clinicopathological features, which could be used to improve cancer diagnosis and treatment in the future. In this study, we analysed LOX expression separately either in the cytoplasm, or in the nucleus, in a series of rectal cancer specimens from the patients participating in a randomized Swedish rectal cancer trial of preoperative radiotherapy (RT) with long-term follow up data [21, 22]. We also charactersied the expression and localisation of LOX in three well-known colon cancer cell lines. We are the first to show LOX (50-kDa and 32-kDa isoform) expression in the nucleus of patient samples and *in vitro* in cancer cells and, more importantly, we identify it as an independent prognostic factor in rectal cancer patients.

## RESULTS

### Cytoplasmic and nuclear expression of LOX in normal mucosa, primary tumour and lymph node metastases

**Figure 1:**
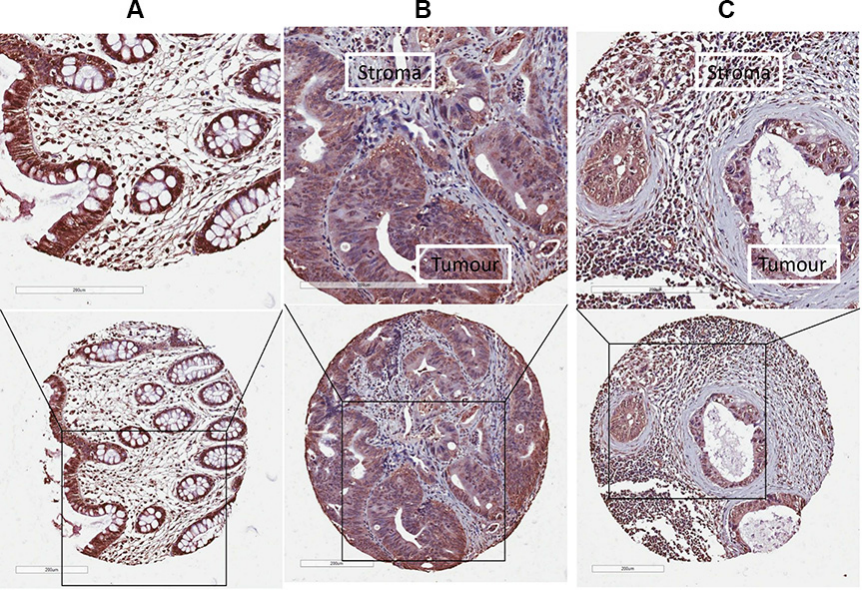
The expression of LOX protein determined by IHC LOX expression was detected both in the cytoplasm and in the nucleus of normal epithelial cells or cancer cells in (**A**) normal mucosa, (**B**) primary tumour, and (**C**) lymph node metastases.

The expression and localisation of LOX was investigated in normal mucosa, primary tumour tissue and lymph node metastases by immunohistochemistry (IHC). LOX expression was detected both in the cytoplasm and in the nucleus of normal epithelial cells and cancer cells (Figure 1). Thus, in subsequent analyses, cytoplasmic and nuclear immunostaining patterns for LOX were scored independently.

**Figure 2:**
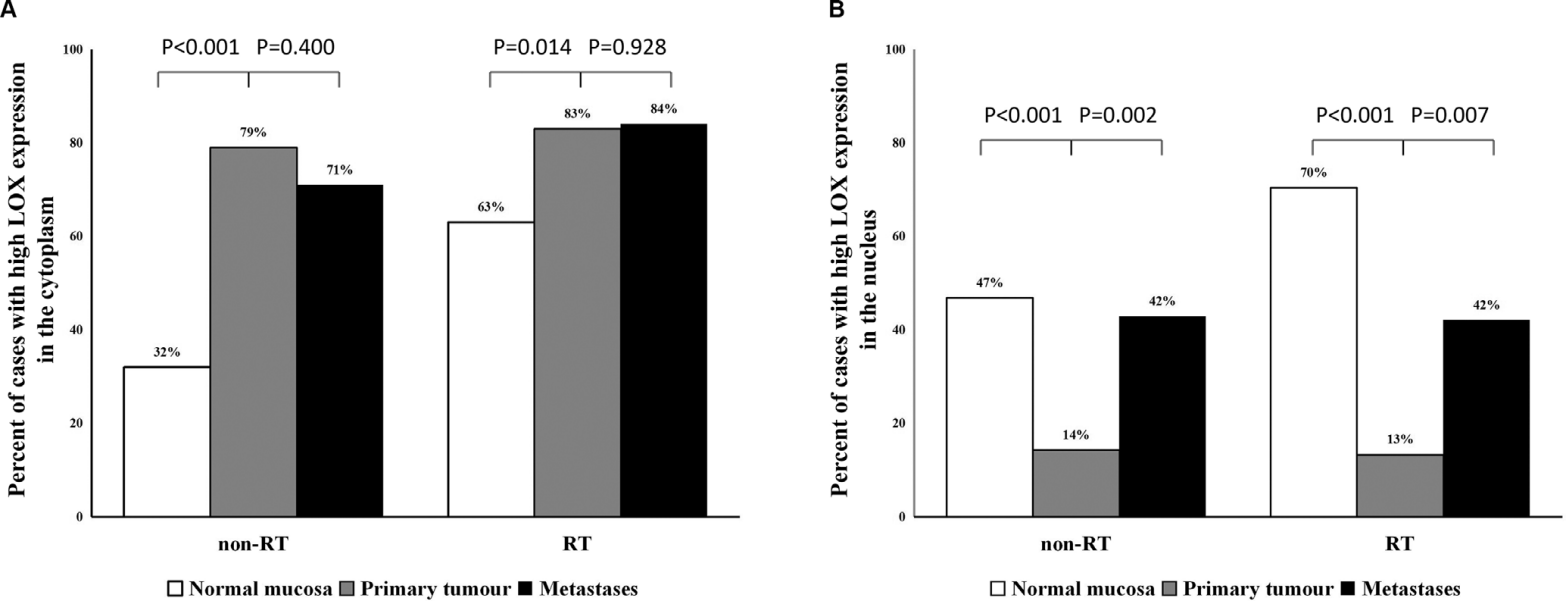
The frequency of high cytoplasmic and nuclear expression of LOX protein (**A**) The percentage of high cytoplasmic LOX expression significantly increased from normal mucosa to primary tumour. (**B**) The LOX expression in the nucleus was significantly decreased in the primary tumour compared with normal mucosa and increased from primary tumour to lymph node metastases.

When examining cytoplasmic immunostaining patterns of LOX, we observed higher levels of LOX expression in primary tumour tissue (79% of 77 (non-RT), and 83% of 60 (RT)) compared to normal mucosa (32% of 62 (non-RT), and 63% of 54 (RT)) for both non-RT (*P* < 0.001) and RT (*P* = 0.014) patients (Figure 2A, Supplementary Figure 1A). There was no significant difference between cytoplasmic LOX immunostaining in primary tumour tissue and lymph node metastases in either group (non-RT/RT) (*P* = 0.400 and *P* = 0.928, respectively). The results show that cytoplasmic LOX is associated with diseased tissue.

In contrast, when analysing nuclear immunostaining patterns, the frequency of high LOX expression was significantly lower in primary tumour (14% of 77, and 13% of 60) than in normal mucosa (47% of 62, and 70% of 54) in both non-RT and RT groups (*P* < 0.001 for both). However, significantly increased expression of LOX in the nucleus was observed in lymph node metastases when compared with primary tumour in both non-RT and RT groups (*P* = 0.002 and *P* = 0.0007, respectively; Figure 2B, Supplementary Figure 1B). Our data show that changing nuclear LOX expression at the primary tumour may be a marker of transition to metastasis. Additional analysis demonstrated that there was no correlation between high cytoplasmic LOX expression and low nuclear LOX expression or between low cytoplasmic expression and high nuclear expression (*P* > 0.05).

Since preoperative RT has been well established as a standard treatment in rectal cancer, we further evaluated the effect of RT on LOX expression and localisation. RT significantly increased the expression of LOX in both the cytoplasm (*P* < 0.001) and nucleus (*P* = 0.010) in normal mucosa whereas no significant difference was found either in primary tumours (*P* = 0.543 for the cytoplasm, and *P* = 0.873 for the nucleus) or in lymph node metastases (*P* = 0.310 for the cytoplasm, and *P* = 0.960 for the nucleus) (Supplementary Figure 2). These results show that although normal epithelial cells respond to RT by increasing LOX expression, in cancer cells the already high expression and localisation patterns of LOX are unaffected by RT.

### The localisation and expression of LOX protein in cancer cell lines

**Figure 3:**
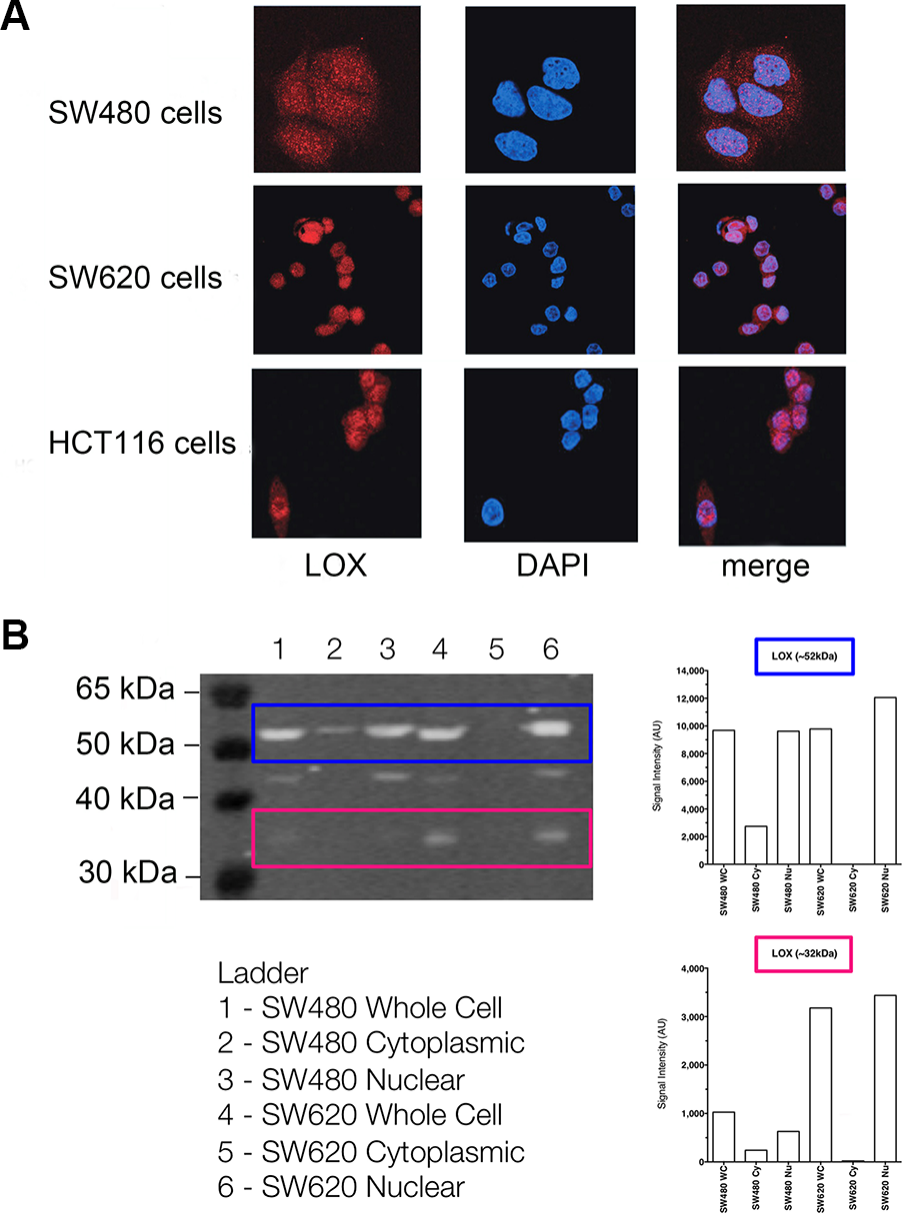
The subcellular localisation of LOX protein in colon cancer cell lines (**A**) Immunofluorescence of LOX expression determined by confocal microscopy in SW480, SW620 and HCT 116 cells. (**B**) Western blot analysis of LOX expression in cytoplasmic and nuclear fractions from SW480 and SW620 cells.

To further confirm the subcellular localisation of LOX, we examined LOX expression in SW480, SW620 and HCT116 cancer cell lines using laser scanning confocal microscopy. In line with the results obtained in patient tumour tissue above, LOX was detected both in the cytoplasm and in the nucleus of all three cell lines (Figure 3A).

Upon examining the expression of LOX by Western blot, we detected a clear and strong band of approximately 52 kDa, which is consistent with the expected size of LOX N-glycosylated proenzyme, as well as a weaker band around 32 kDa consistent with the mature enzyme form. The both bands were detected in whole cell and nuclear extracts of SW480 and SW620 cells (Figure 3B).

The two bands were weak in cytoplasmic extracts of SW480 cells and hardly detected in cytoplasmic extracts of SW620 cells. In nuclear extracts, both forms were found increased in the metastatic cell line SW620 compared to the parental non-metastatic cancer cell line SW480. The presence of 46kDa non-glycosylated proenzyme form was also observed in whole cell and nuclear extracts of SW480 and SW620 cells. Radiation did not affect LOX protein expression (neither localisation nor expression) in the two cell lines, consistent with the results obtained in the cancer tissues above.

### Relationship of LOX protein expression with clinicopathological variables

**Figure 4:**
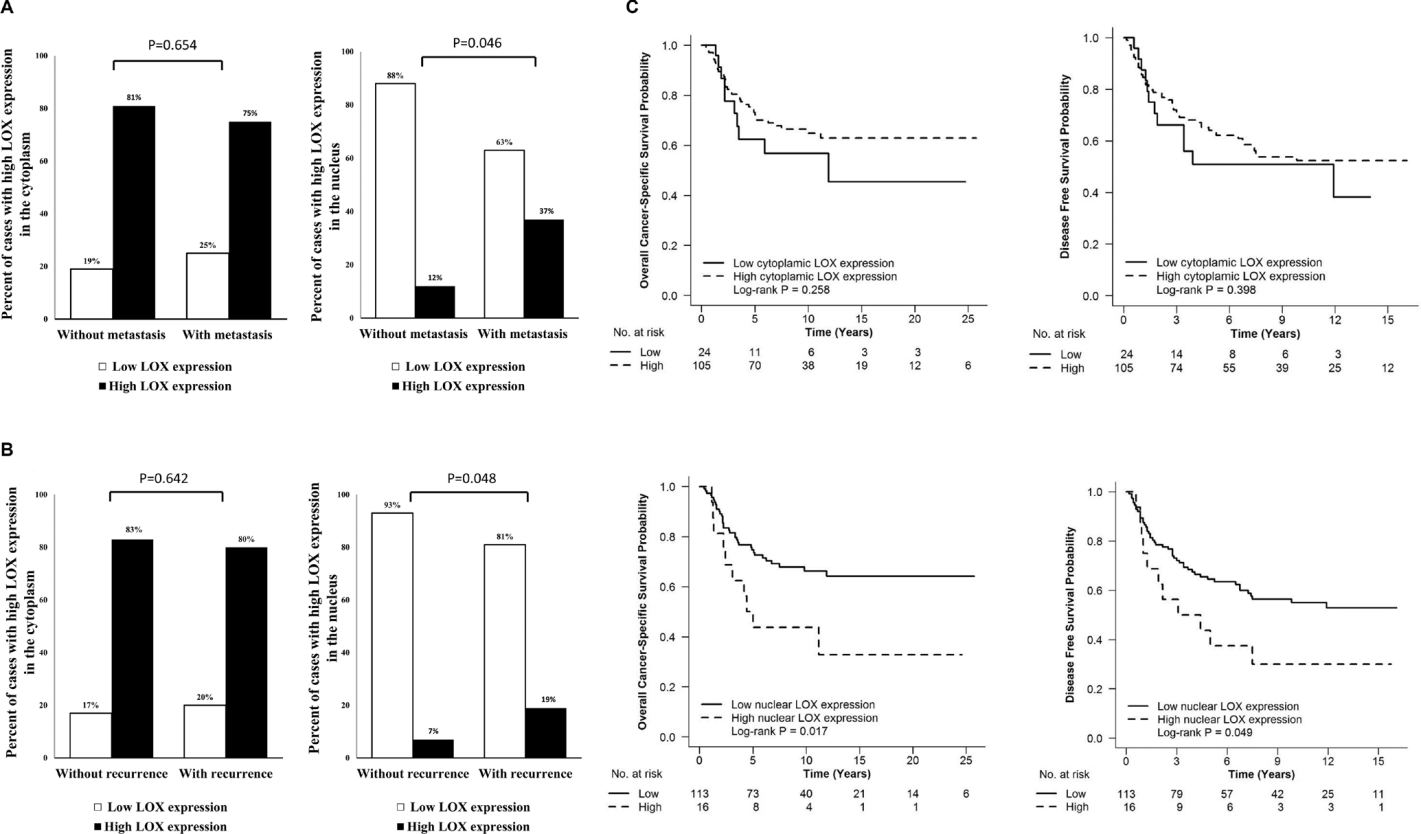
The relationship between LOX expression in primary tumour and distance metastasis, total recurrence and survival High nuclear LOX expression was related to high rate of distant metastasis (**A**) and total recurrence (**B**) compared with low nuclear LOX expression in primary tumour. Patients with high nuclear LOX expression had poor OS (**C**) and poor DFS (**C**).

Since RT had no influence on LOX expression in primary tumour; we combined non-RT and RT groups together for further analysis. High nuclear LOX expression was correlated to a higher rate of distant metastasis and total recurrence (local and/or distant recurrence) compared with lower nuclear LOX expression (*P* = 0.046, and *P* = 0.048, respectively; Figure 4A and 4B, Supplementary Table 1). There was no correlation of cytoplasmic LOX expression to gender, age, tumour node metastasis (TNM) stage, distant metastasis, total recurrence or differentiation (*P* > 0.05, Supplementary Table 1).

In survival analysis, high nuclear LOX expression in stage I to III cancers was correlated to poor overall cancer-specific survival (OS) (*P* = 0.017, Figure 4C) and disease free survival (DFS) (*P* = 0.049, Figure 4C). In multivariable analysis, the significance still remained after adjusting for gender, age, TNM stage, differentiation and RT (*P* = 0.003 for OS, and *P* = 0.004 for DFS, Table 1). However, cytoplasmic staining of LOX was not related to either DFS or OS (*P* > 0.05, Figure 4C).

**Table 1:** Multivariable analysis of nuclear LOX expression associated with survival of rectal cancer patients

Variables^a^	OS			DFS		
	HR	95% CI	*P*-value	HR	95% CI	*P*-value
Nuclear LOX expression (High vs. Low)	2.975	1.440–6.148	0.003	2.735	1.376–5.438	0.004
Gender (Female vs. Male)	0.644	0.345–1.204	0.168	0.706	0.409–1.218	0.211
Age (≥ 66 vs.< 66)	2.169	1.127–4.175	0.021	1.647	0.951–2.853	0.075
TNM stage (III vs. I + II)	8.142	4.035–16.431	< 0.001	7.359	4.089–13.242	< 0.001
Differentiation (Poorly vs. Well + Moderately)	0.807	0.389–1.676	0.566	0.620	0.309–1.247	0.180
RT (With vs. Without)	0.900	0.489–1.656	0.734	0.812	0.477–1.385	0.445

^a^Significance was analysed by Cox regression model

### Relationship of LOX protein expression with biological factors

We further analysed the relationship between LOX expression and biological factors previously investigated within the same patient cohort. There was a significant positive correlation between cytoplasmic LOX expression with survivin, a negative regulator of apoptosis (*P* = 0.029, Table 2). Nuclear LOX expression in the primary tumour was also positively correlated with phospho-NF-κB p65 (Serine 536) expression (*P* < 0.001), and showed a weak, albeit statistically significant positive correlation with Ki-67 expression (*P* = 0.05).

**Table 2:** LOX expression in the primary rectal cancer in relation to biological variables

Variables	LOX expression					
	Cytoplasmic			Nuclear		
	Low (%)	High (%)	*P*-value	Low (%)	High (%)	*P*-value
NF-κB			0.388			**< 0.001**
Low	15 (17)	74 (83)		84 (94)	5 (6)	
High	11 (23)	37 (77)		34 (71)	14 (29)	
Ki-67			0.258			**0.050**
Low	7 (13)	45 (87)		49 (94)	3 (6)	
High	12 (22)	43 (78)		45 (82)	10 (18)	
Survivin			**0.029**			0.215
Low	14 (22)	50 (78)		57 (89)	7 (11)	
High	0 (0)	18 (100)		14 (78)	4 (22)	

## DISCUSSION

In this study, we demonstrated the presence of nuclear localised LOX expression, in addition to cytoplasmic expression, in patient material. We have shown that nuclear LOX expression was significantly increased in lymph node metastases compared to primary tumours, and that this high nuclear expression was correlated with distance metastasis. Thus our findings suggest that LOX localisation may be an important marker of transition to metastasis. Altered LOX localisation was further confirmed by confocal microscopy and Western blot in cancer cell lines. Furthermore, for the first time, we showed by multivariable analysis that nuclear LOX, not cytoplasmic LOX, was a robust and independent prognostic factor in rectal cancer patients. Nuclear LOX also proved to be correlated with other biological factors (such as NF-κB) known to be associated with patient survival.

Previously, few reports have shown that LOX enzyme expression could be detected in the nucleus of normal tissue. Kagan *et al*. first documented the presence and retained catalytic activity of LOX mature enzyme within the nuclei of rat vascular smooth muscle cells and NIH 3T3 fibroblasts [23]. Later, it was demonstrated that exogenous mature LOX enzyme could enter the cells and concentrate within the nuclei [24]. Here for the first time, our results demonstrated the existence of LOX proenzyme (50kDa) and mature enzyme (32kDa) within the nuclei of colon cancer cells. The precise role of nuclear LOX has yet to be determined. Some experimental data have shown that LOX catalyses the oxidation of residues in histones H1 and H2, which are known to control global chromatin compaction and the expression of selected genes [6, 14, 25].

Both LOX down- and up-regulation has been described in CRC as well as other cancers, initially, decreased LOX mRNA expression levels were reported in CRC patients with non-metastatic disease [26]. However, work from both us and other groups observed an association of LOX overexpression and CRC progression [15, 20, 27, 28]. One important issue is, in these studies, that the authors did not profile the nuclear localisation of LOX protein. In the present study, our data showed the increased expression of LOX in the nucleus of the matched patient lymph node metastases compared to primary tumour. Furthermore, high nuclear LOX expression in primary tumours was also shown to be significantly associated with high frequency of metastatic disease. In support of this, we also observed elevated LOX expression in the nucleus of the metastatic colon cancer cell line (SW620), a cell line derived from a lymph node metastases taken from the same patient as the isogenically matched non-metastatic colon cancer cell line (SW480). In addition to this, our previous work has shown that overexpression of LOX in the non-metastatic SW480 cell line leads to increased tumour cell proliferation and metastasis both *in vitro* and *in vivo*. In contrast, knocking down of LOX expression in the SW620 metastatic cell line significantly reduces tumour cell proliferation and metastasis [15, 20]. Moreover, our latest results show that a high expression of LOX in primary tumour cells leads to tumour-driven pre-metastatic bone lesions whereas inhibition of LOX abrogates this process [19]. Taken all of these results together, it has been highly implied that nuclear localisation of LOX plays an important role in the process of cancer metastasis.

Our data further show that high nuclear expression of LOX was positively correlated with poor survival in rectal cancer patients in multivariable analysis. Recently, LOX has been clinically validated as a prognostic biomarker for metastatic head and neck cancer [10]. This work complements our findings and, furthermore, LOX has also been proposed to offer similar prognostic value in oral and oropharyngeal squamous carcinoma, whereby high expression is correlated with poor disease-free and overall survival [11]. Importantly though, despite implicating overall LOX expression, neither of these papers profiled the specific cellular localisation of LOX in tumour tissues, making this the first report to do so.

In the present study, our data showed that nuclear rather than cytoplasmic LOX protein expression in the primary rectal cancer was positively correlated with phospho-NF-κB p65 expression. It was originally shown that the LOX-PP could inhibit NF-κB activity and induced phenotypic reversion of cancer cells [17, 29]. However, a recent paper has reported that LOX expression displayed a positive correlation with NF-κB p65 expression in granulosa cells of ovarian tissue [30]. Notably, the antibody used in their study recognized both the LOX proenzyme and mature enzyme, yet they further demonstrated that NF-κB could up-regulate LOX gene expression by binding to its promoter. However, the authors did not assess the localisation of LOX protein. Since nuclear LOX presence may be involved in affecting specific gene expression patterns, we speculate that there may exist a positive LOX-NF-κB feedback loop promoting cancer metastasis in CRC. LOX enzyme may activate NF-κB in the nucleus, and activated NF-κB thus in turn further increase LOX expression. Nevertheless, it is too early to speculate on the detailed mechanisms underlying the relationship between LOX and NF-κB, and as such requires further investigation.

We also analysed whether RT affected the LOX expression and localisation in patient samples and cell lines since preoperative RT is the standard treatment for rectal cancer patients. Our results show that LOX expression both in the cytoplasm and in the nucleus increased in normal mucosa samples after RT. This is in line with other work which has shown increases in LOX expression in radiation lung injury [31]. Our previous work has also shown that the activity of increased LOX expression induced in lung tissue by radiation, will enhance metastatic colonisation of cells [32]. Although it has been demonstrated that radiation can induce LOX secretion in several types of tumour cells [33], our data did not show any evidence of significant differences in either cytoplasmic or nuclear LOX expression related to RT in either patient samples or *in vitro* cancer cells. This suggests that CRC tumour cells, which already exhibit elevated LOX expression, do not upregulate LOX further in response to RT. This allows us to use primary tumour LOX expression and localisation as a robust prognostic indicator for patients at high risk of relapse independent of whether they received preoperative RT.

In conclusion, this study demonstrated that LOX expression in the nucleus is a promising prognostic biomarker in rectal cancer patients. It sheds a new light on better understanding the complex and crucial role of LOX in cancer beyond its well-known extracellular matrix-modifying function.

## MATERIALS AND METHODS

### Patient material

The patients were from the South-East Swedish Health Care region and participated in the randomized Swedish rectal cancer trial of preoperative RT between 1987 and 1990 [21]. Each participant signed the informed consent. The patient cohort included 137 primary rectal adenocarcinomas, 116 normal mucosa specimens (104 corresponding to the primary tumour, i.e., normal mucosa and primary tumour from the same patient) that were histologically free from cancer and taken from the margin of distant surgical resection, and 47 lymph node metastases (42 corresponding to the primary tumour, also in the radiation field). Of the 137 patients (average age, 66.4 years), 77 underwent surgery alone and 60 received RT followed by tumour resection. RT was given with 25 Gy in 5 fractions within a median of 7 days (range, 4–12 days). Surgery was then carried out in a median of 3 days (range, 0–11 days) after RT. None of the patients received preoperative or adjuvant chemotherapy. The mean follow-up period was 100 months (range, 0–309 months), and information on local and distant recurrence, DFS and OS were obtained from patient medical records. The characteristics of the patients and tumours are presented in Supplementary Table 2.

### Human colon cancer cell lines

The SW480 and SW620 colon cancer cell lines were obtained from the American Type Culture Collection (Manassas, VA). SW480 cell line was established from primary adenocarcinoma of the colon, and the SW620 from metastatic lymph node, taken from the same patient one year later. The cells were kept at 37°C and 5% CO2 in Eagle's MEM (Sigma-Aldrich, St Louis, MO) supplemented with 10% heat-inactivated fetal bovine serum albumin (GIBCO, Invitrogen, Paisley, UK) and 1% Penicillin-streptomycin (GIBCO). The HCT116 colon cancer cell line was obtained from the core cell center (Johns Hopkins University, Baltimore, MD), and was maintained in McCoy's 5A medium (Sigma-Aldrich) supplemented with 10% heat-inactivated fetal bovine serum albumin (GIBCO) and 1% Penicillin-streptomycin (GIBCO) and kept in the same manner as SW480 and SW620. All cell lines were routinely tested as negative for mycoplasma.

### Radiation of cancer cell lines

All experiments were carried out in biological triplicate. Briefly, 1 × 10^6^ SW480 or SW620 cells were plated into standard 85 mm tissue culture plates 24 hours prior to radiation treatment. Cells were irradiated using the Faxitron CP160 X-Ray irradiator with either 2Gy or 5Gy at a rate of 0.7Gy per minute. Control cells were mock-irradiated under the same conditions.

### IHC

IHC staining for LOX expression was done on 4 μm tissue microarray sections from paraffin-embedded surgical specimens. Sections were deparaffinized by immersing the slides twice in 100% xylene at room temperature for 10 minutes each. This was followed by incubating twice in 100% ethanol for 10 minutes each, and rehydrating with decreasing concentrations of ethanol (90% and 70%; vol/vol in water, 10 minutes each) before a final 5-minute incubation in water. Antigen retrieval was carried out in a target retrieval citrate buffer (pH 6.0) (Dako, Glostrup, Denmark) at 95°C for 15 minutes. The sections were allowed to cool for 15 minutes and rinsed with phosphate-buffered saline (PBS). The sections were incubated in 3% H_2_O_2_-methanol for 5 minutes to block the activity of endogenous peroxidase. After being washed in PBS, the sections were incubated with protein block (Dako) for 10 minutes to reduce nonspecific background staining. The sections were incubated with the anti-LOX polyclonal rabbit antibody (synthesized by OpenBiosystems which targets a conserved peptide sequence from the active site of human and mouse proteins and has been shown not to bind other LOX family members [35]) in a 1:50 dilution with antibody diluent buffer overnight. After being washed in PBS, the sections were incubated with a secondary antibody, Envision System Labelled Polymer-HRP Anti-Rabbit (Dako) for 25 minutes. The sections were rinsed in PBS before reacting with Liquid DAB+ (Dako) to produce coloration. Finally, the sections were lightly counterstained with hematoxylin.

The immunostaining was scored by two independent observers based on the intensity and localisation without information of the patients. The intensity in epithelial cells or tumour cells was scored as negative, weak (light yellow), moderate (yellow brown), and strong staining (brown). The staining patterns were graded as cytoplasmic or nuclear. In the case of discrepant scoring results, a consensus score was reached after re-evaluation. For statistical analyses, negative and weak-stained cases were considered as low-expressing group, and moderate and strong staining as high-expressing group. The expression of NF-κB, Ki-67 and survivin was determined by IHC at our laboratory on the same patient samples as in the present study. The used cutoff points were the same as in the corresponding publications [34, 35, 36].

### Confocal microscopy

Cells were seeded on coverslips and allowed to adhere overnight in 6-well plates, then fixed with 4% paraformaldehyde, and permeabilized with 0.5% triton-x-100 in PBS. The cells were blocked in blocking buffer for 30 minutes, and then stained with the primary antibody (described above) in a 1:50 dilution and incubated at room temperature for 1.5 hours. The reaction was stopped by adding wash buffer and rinsing 3 times. Cells were then incubated with anti-rabbit Alexa Fluor 488 conjugated secondary antibody (Thermo Fisher Scientific, Waltham, MA) for 30 minutes at 37°C. Coverslips with cells were fixed on glasses by adding mounting medium with Diamidino-2-phenylindole (DAPI) (Olink Bioscience, Uppsala, Sweden). Prepared cells were analysed in upright Zeiss Axio Imager with LSM 700 confocal microscope (Zeiss, Germany) using a 63× oil immersion objective.

### Western blot

Cytoplasmic and nuclear extracts were prepared as follows. After 24 hours post radiation, cells were scraped into 1 mL PBS, and fractionated using NP-40 buffer to generate total, cytoplasmic and nuclear fractions. Fractions were mixed with Laemmli buffer and sonicated before SDS-PAGE immunoblotting for LOX. The protein concentration was determined by the colorimetric Bradford protein assay reagent. Equal amounts of protein were loaded, separated by electrophoresis, and transferred to PVDF membrane. Membranes were blocked with 5% milk powder in TBS containing 0.1% Tween-20 for 1 hour at room temperature and incubated with the primary antibody (described above) (1:200) overnight at 4°C. The membranes were washed and subsequently incubated with the secondary HRP-conjugated polyclonal goat anti-rabbit (1:10000, DAKO) for 1 hour. Protein bands were detected using ECL plus Western Blotting Detection System (Amersham Bioscience/GE Healthcare, Piscataway, NJ). Anti-β-actin (1: 5000, Cell Signaling Technology, Danvers, MA) was used as a loading control.

### Statistical analyses

All statistical analyses were carried out by using STATISTICA software package (version 7.0; STATSOFT Inc., Tulsa, OK). McNemar's or Person χ^2^ test was used to examine the significance of the differences in LOX expression among normal mucosa, primary tumour, and lymph node metastases, as well as the association of LOX expression with clinicopathological or biological variables. The relationship of LOX expression with survival was tested by using Kaplan–Meier analysis (Log rank test) and Cox proportional hazards regression analysis (likelihood ratio test). All tests were two sided and *P*-values of < 0.05 were considered as significant.

## SUPPLEMENTARY FIGURES AND TABLES


