# Supplementary material for: Nuclear expression of lysyl oxidase enzyme is an independent prognostic factor in rectal cancer patients
Source: Oncotarget. 2016 May 26;8(36):60015–24. doi: 10.18632/oncotarget.9623 (PMC5601118; doi:10.18632/oncotarget.9623)
Supplement: Supplementary file 1 [file oncotarget-08-60015-s001.pdf]

Nuclear expression of lysyl oxidase enzyme is an independent prognostic factor in rectal cancer patients

Supplementary Materials

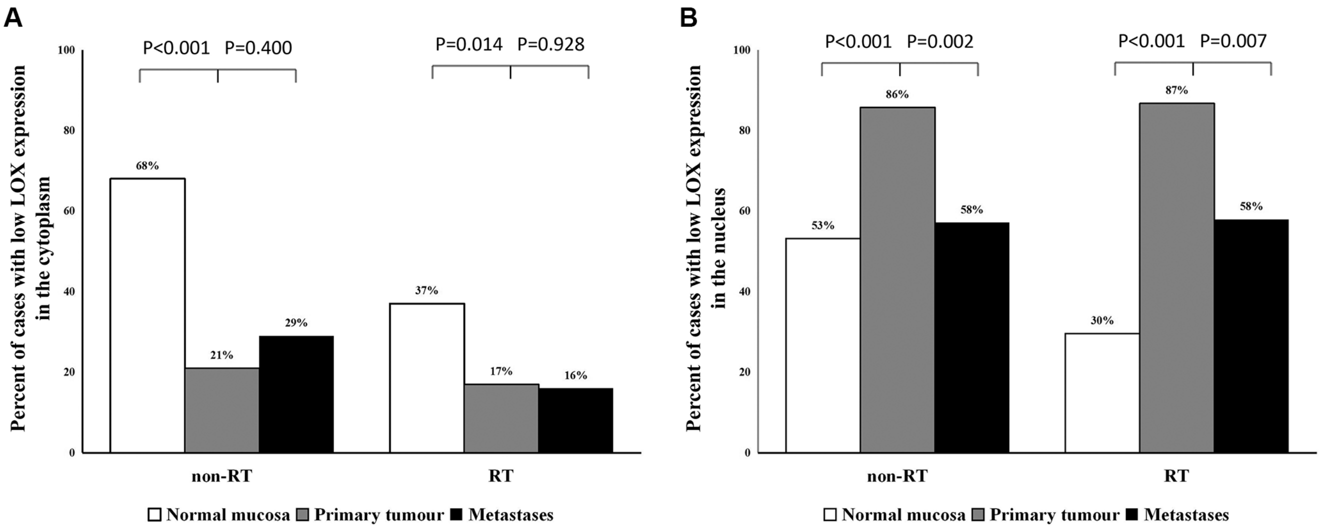

Supplementary Figure S1: The frequency of low cytoplasmic and nuclear expression of LOX protein. (**A**) The percentage of low cytoplasmic LOX expression decreased from normal mucosa to primary tumour significantly. (**B**) The frequency of low LOX nuclear expression was increased significantly in the primary tumour compared with normal mucosa and decreased from primary tumour to lymph node metastases.

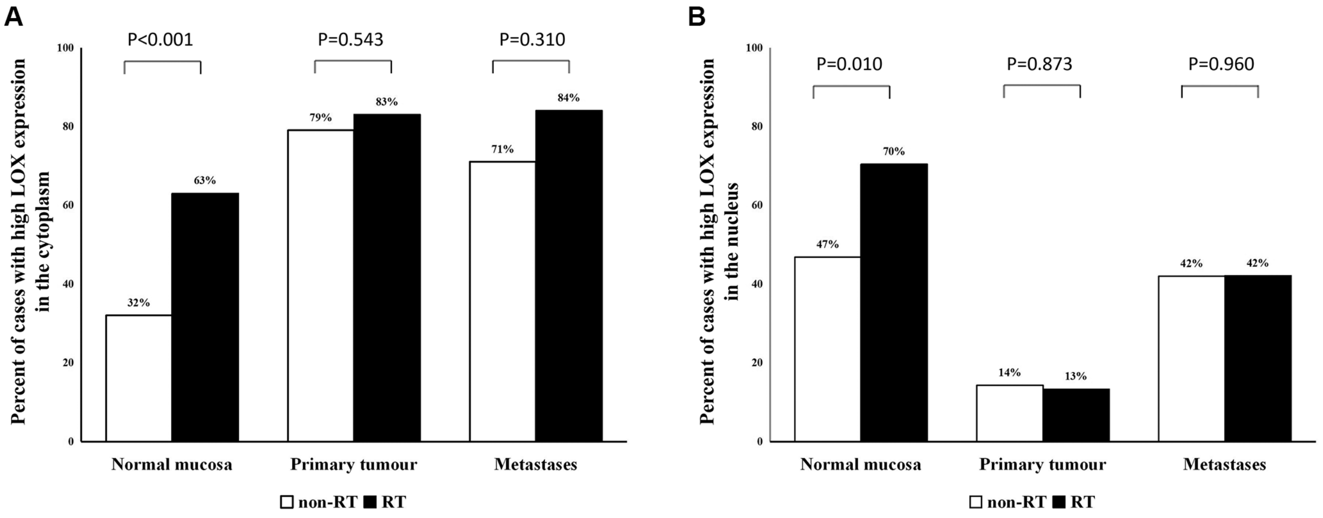

Supplementary Figure S2: The influence of RT on LOX expression/localisation. Both in the cytoplasm (**A**) and in the nucleus (**B**), when compared with non-RT group, the frequency of highly expressed LOX in RT was significantly increased in normal mucosa sample, whereas no significant difference was found either in primary tumour or in lymph node metastases.

Supplementary Table S1: LOX expression in the primary rectal cancer in relation to clinicopathological variables

Variables	LOX expression					
	Cytoplasmic			Nuclear		
	Low (%)	High (%)	*P*-value	Low (%)	High (%)	*P*-value
Gender			0.220			0.451
Male	13 (16)	70 (84)		70 (84)	13 (16)	
Female	13 (24)	41 (76)		48 (89)	6 (11)	
Age (years)			0.228			0.134
< 66	7 (14)	44 (86)		41 (80)	10 (20)	
≥ 66	19 (22)	67 (78)		77 (90)	9 (10)	
TNM stage			0.065			0.094
I	10 (28)	26 (72)		34 (94)	2 (6)	
II	2 (5)	37 (95)		32 (82)	7 (18)	
III	12 (22)	42 (78)		47 (87)	7 (13)	
IV	2 (25)	6 (75)		5 (62)	3 (38)	
Differentiation			0.679			0.654
Well	0 (0)	3 (100)		3 (100)	0 (0)	
Moderately	21 (20)	85 (80)		90 (85)	16 (15)	
Poorly	5 (18)	23 (82)		25 (89)	3 (11)	
Distant metastasis			0.654			0.046
No	24 (19)	105 (81)		113 (88)	16 (12)	
Yes	2 (25)	6 (75)		5 (63)	3 (37)	
Recurrence[a]			0.642			0.048
No	12 (17)	58 (83)		65 (93)	5 (7)	
Yes	12 (20)	47 (80)		48 (81)	11 (19)	
Local recurrence[a]			0.585			0.847
No	19 (18)	88 (82)		94 (88)	13 (12)	
Yes	5 (23)	17 (77)		19 (86)	3 (14)	
Distant recurrence[a]			0.974			0.092
No	15 (19)	66 (81)		74 (91)	7 (9)	
Yes	9 (19)	39 (81)		39 (81)	9 (19)	

[a]Accounting only for tumour stage I–III.

Supplementary Table S2: Characteristics of patients and tumours

Characteristics	Non-RT<br>*n* (%)	RT<br>*n* (%)	*P* value
Gender			0.351
Male	44 (57)	39 (65)	
Female	33 (43)	21 (35)	
Age (years)			0.920
< 66	29 (38)	22 (37)	
≥ 66	48 (62)	38 (63)	
TNM stage			0.227
I	21 (28)	15 (25)	
II	17 (22)	22 (36)	
III	35 (45)	19 (32)	
IV	4 (5)	4 (7)	
Differentiation			0.723
Well	2 (3)	1 (2)	
Moderately	61 (79)	45 (75)	
Poorly	14 (18)	14 (23)	
Number of tumours			0.426
Single	65 (84)	49 (82)	
Multiple[a]	10 (13)	11 (18)	
Unknown	2 (3)	0 (0)	
Resection Margin			0.597
Negative	73 (95)	58 (97)	
Positive	4 (5)	2 (3)	

[a] Other colorectal cancer and/or other tumour before present rectal cancer.
